# Supplementary material for: Structural Luneburg lens for broadband cloaking and wave guiding
Source: Sci Rep. 2020 Sep 3;10:14556. doi: 10.1038/s41598-020-71124-5 (PMC7471698; doi:10.1038/s41598-020-71124-5)
Supplement: Supplementary file 1 — Supplementary information [file 41598_2020_71124_MOESM1_ESM.pdf]

Structural Luneburg Lens for Broadband Cloaking and Wave Guiding

Liuxian Zhao[a], Miao Yu[a,b,*]

[a] Institute for Systems Research, University of Maryland, College Park, MD, 20742, USA

[b] Department of Mechanical Engineering, University of Maryland, College Park, Maryland 20742, USA

*Author to whom correspondence should be addressed: mmyu@umd.edu

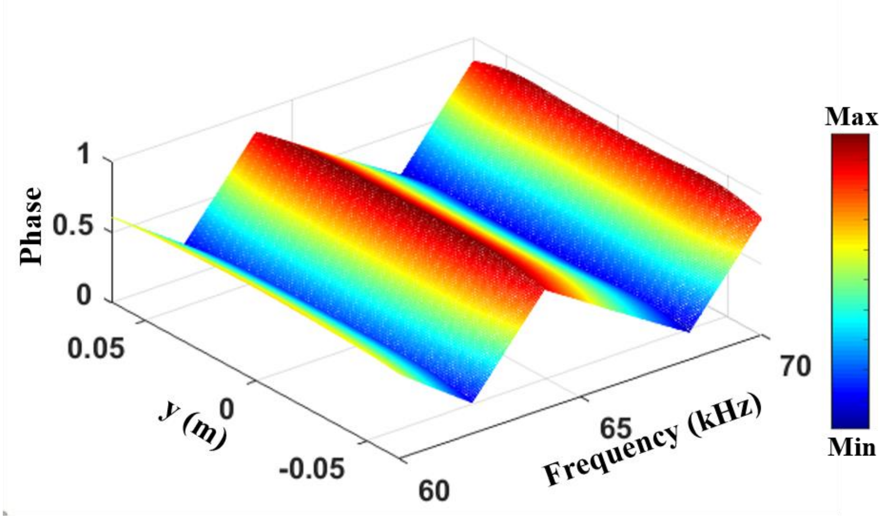

Figure S1: Normalized phase distribution over the frequency range of 60 - 70 kHz for acoustic cloaking. The mesh size was half of that used for obtaining Figure 2 (g).

In order to further investigate the performance of the SLL cloak and the SLL waveguide, a plate with a constant thickness (without SLLs) was used in simulations for comparison. The schematics of thin plates without and with the SLL cloak are shown in Figure S2 (a) and (b), respectively. The Numerical simulation results of waveform without and with SLL cloak at the frequency of 40 kHz are shown in Figure S2 (c) and (d). The displacement amplitudes were obtained at points P1 and P2, which were used to calculate the attenuation. The attenuation is defined as $\alpha = d(\mathrm{P2})/d(\mathrm{P1})$ and plotted in Figure S2 (e), where d represents the displacement. This figure clearly shows that the vibration displacement is reduced over a broad frequency range from 25 – 70 kHz with a minimum attenuation value of 0.39 at the frequency of 47 kHz.

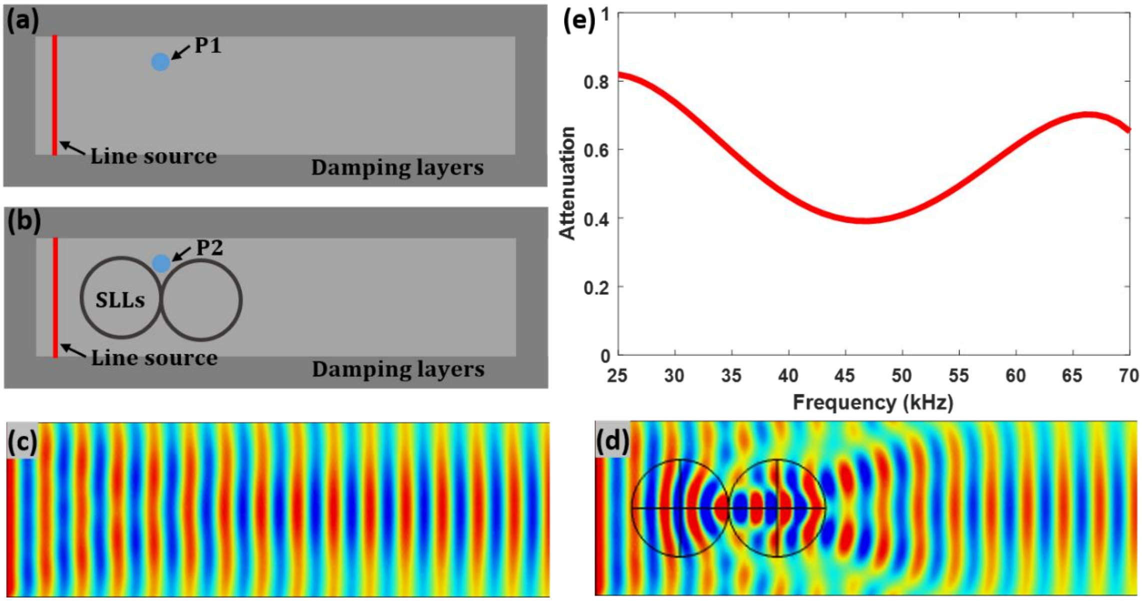

Figure S2: Structural cloaking performance in terms of steady state response. (a) and (b) Schematics of plates without and with SLL cloak, (c) and (d) Numerical simulation results of waveform without and with SLL cloak at the frequency of 40 kHz, (e) Attenuation as a function of frequency.

For the structural waveguide, the schematics of thin plates without and with the SLL waveguide are shown in Figure S3 (a) and (b), respectively. The displacement amplitude values at points P3, P4 and P5 were obtained for the calculation of the gain and the transmission. The gain is defined as $g = d(\mathrm{P4})/d(\mathrm{P3})$, and the transmission is $t = d(\mathrm{P4})/d(\mathrm{P5})$,where d represents the displacement. The gain and transmission as a functional of frequency are plotted in Figures S3(c) and (d). Compared with the case without the SLL waveguide, the vibration displacement is clearly enhanced over a broadband frequency range from 25 – 70 kHz with a gain ranging from 3 to 5 (maximum gain of 5 at 47 kHz). In addition, the transmission is between 0.76 to 0.93 over a broad frequency range of 25 – 70 kHz, which indicates that the SLLs exhibit a good performance for waveguide.

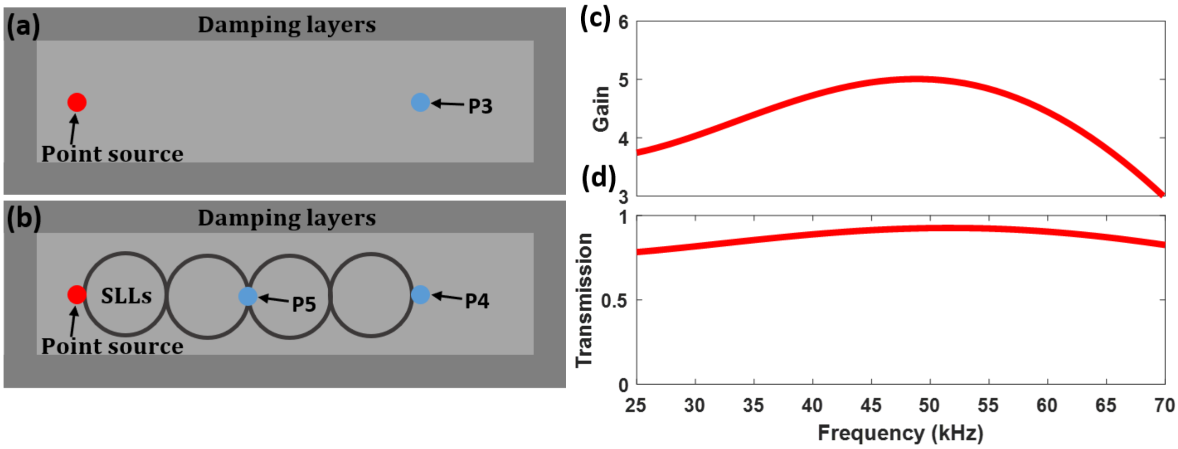

Figure S3: Structural waveguide performance in terms of steady state response. (a) Schematics of plates without and with SLL waveguide, (c) and (d) Gain (displacement ratio between P4 and P3) and transmission (displacement ratio between P4 and P5) spectra.

For the time domain simulations, a signal with a 3-count Hanning-windowed tone burst at f = 40 kHz was used at the input signal of the line source and the point source, as shown in Figure S4.

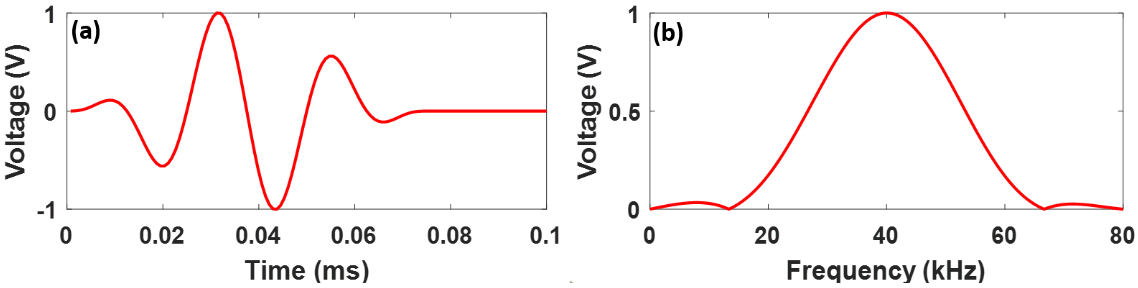

Figure S4: Input signal for the time domain analysis: (a) time domain and (b) frequency domain.
